# The natural history of adult pulmonary Langerhans cell histiocytosis: a prospective multicentre study

**DOI:** 10.1186/s13023-015-0249-2

**Published:** 2015-03-14

**Authors:** Abdellatif Tazi, Constance de Margerie, Jean Marc Naccache, Stéphanie Fry, Stéphane Dominique, Stéphane Jouneau, Gwenaël Lorillon, Emmanuelle Bugnet, Raphael Chiron, Benoit Wallaert, Dominique Valeyre, Sylvie Chevret

**Affiliations:** Assistance Publique-Hôpitaux de Paris, Hôpital Saint-Louis, Centre National de Référence de l’histiocytose Langerhansienne, Service de Pneumologie, 1 Avenue Claude Vellefaux, 75475 Paris Cedex 10, France; Univ Paris Diderot, Sorbonne Paris Cité, U1153 CRESS, Biostatistics and Clinical Epidemiology research team, Paris, France; Assistance Publique-Hôpitaux de Paris, Service de Radiologie, Hôpital Saint-Louis, Paris, France; Assistance Publique-Hôpitaux de Paris, Service de Pneumologie, Hôpital Avicenne, Bobigny, France; Service de Pneumologie et Immuno-allergologie, Centre de compétence des maladies pulmonaires rares, Hôpital Calmette, Lille, France; Département de Pneumologie, Hôpital Charles Nicolle, Rouen, France; IRSET UMR 1085, Université de Rennes 1; Service de Pneumologie, Hôpital Pontchaillou, Rennes, France; Département de Pneumologie, Hôpital Arnaud de Villeneuve, Montpellier, France; Université Lille 2, Lille, France; Université Paris 13, Sorbonne Paris Cité; Assistance Publique-Hôpitaux de Paris, Service de Pneumologie, Hôpital Avicenne, Bobigny, France; Assistance Publique-Hôpitaux de Paris; Hôpital Saint-Louis, Service de Biostatistique et Information Médicale, Paris, France

**Keywords:** Langerhans cell histiocytosis, Lung function, High resolution computed tomography, Smoking, Outcome, Health quality of life

## Abstract

**Background:**

The natural history of pulmonary Langerhans cell histiocytosis (PLCH) has been unclear due to the absence of prospective studies. The rate of patients who experience an early progression of their disease is unknown. Additionally, conflicting effects of smoking cessation on the outcome of PLCH have been reported.

**Methods:**

In this prospective, multicentre study, 58 consecutive patients with newly diagnosed PLCH were comprehensively evaluated over a two-year period. Our objectives were to estimate the incidence of early progression of the disease and to evaluate the impact of smoking status on lung function outcomes. Lung function deterioration was defined as a decrease of at least 15% in FEV_1_ and/or FVC and/or DL_CO_, compared with baseline values. At each visit, smoking status was recorded based on the patients’ self-reports and urinary cotinine measurements that were blinded for the patients. The cumulative incidence of lung function outcomes over time was estimated using the non-parametric Kaplan-Meier method. Multivariate Cox models with time-dependent covariates were used to calculate the hazards ratios of the lung function deterioration associated with smoking status with adjustment for potential confounders.

**Results:**

The cumulative incidence of lung function deterioration at 24 months was 38% (22% for FEV_1_ and DL_CO_, and 9% for FVC). In the multivariate analysis, smoking status and PaO_2_ at inclusion were the only factors associated with the risk of lung function deterioration. The patients’ smoking statuses markedly changed over time. Only 20% of the patients quit using tobacco for the entire study period. Nevertheless, being a non-smoker was associated with a decreased risk of subsequent lung function deterioration, even after adjustment for baseline predictive factors. By serial lung computed tomography, the extent of cystic lesions increased in only 11% of patients.

**Conclusions:**

Serial lung function evaluation on a three- to six-month basis is essential for the follow-up of patients with recently diagnosed PLCH to identify those who experience an early progression of their disease. These patients are highly addicted to tobacco, and robust efforts should be undertaken to include them in smoking cessation programs.

**Trial registration:**

ClinicalTrials.gov: No: NCT01225601.

**Electronic supplementary material:**

The online version of this article (doi:10.1186/s13023-015-0249-2) contains supplementary material, which is available to authorized users.

## Background

Pulmonary Langerhans cell histiocytosis (PLCH) is a rare cystic disorder of unknown origin that occurs in young adult smokers [1-3]. The disease can resolve spontaneously, remain stable, or progress to respiratory failure with severe pulmonary hypertension (PH), requiring lung transplantation [1,2,4].

The natural history of PLCH is unclear due to a lack of prospective studies. In a long-term, multicentre, retrospective study, we found that lung function had deteriorated in approximately half of patients during the five years of follow-up [5]. We also found that a subgroup of patients experienced a dramatic decline in their forced expiratory volume in 1 second (FEV_1_) early after the diagnosis of their disease [5].

The triggering role of smoking in PLCH has been highlighted by the finding that most children with systemic LCH who develop lung involvement in adolescence or adulthood begin smoking before this event [6]. Smoking cessation is an essential goal for these patients, but conflicting effects of smoking cessation on the outcome of the disease have been reported, particularly because the smoking statuses of the patients have been based on self-reports, and no surrogate markers have been used to ascertain smoking cessation [4,5,7-12]. Furthermore, the smoking status of the patients during follow-up has not been assessed in these retrospective studies.

The creation of the Reference Centre for Langerhans Cell Histiocytosis provided a unique opportunity to conduct a longitudinal, prospective study in a cohort of patients with newly diagnosed PLCH, who were comprehensively evaluated over time. The main objectives of this study were the following: 1) to estimate the incidence of progression early in the course of the disease; and 2) to rigorously assess the smoking status of the patients during follow-up and to seek an association between smoking status and subsequent lung function outcomes.

## Methods

### Study design

This prospective, multicentre study was conducted by the French National Reference Centre for Langerhans Cell Histiocytosis, in collaboration with six hospital pulmonary departments. The inclusion period was from May 2006 to April 2009. The study protocol was approved by the appropriate ethics committee in February 2006 and was registered with www.clinicaltrials.gov (NCT01225601). The study was funded by the French Ministry of Health and the Delegation for Clinical Research of the Assistance Publique-Hôpitaux de Paris. The sponsors had no role in the design, conduct, or data analysis of the study.

### Study subjects

Consecutive patients 18 years of age or older who were referred for PLCH to the participating centres were considered eligible, provided they received no treatment for their disease. The diagnosis of PLCH either was histologically confirmed or was based on the following: 1) an appropriate clinical setting, 2) a typical lung high-resolution computed tomography (HRCT) showing the combination of nodules, cavitated nodules and thick- and thin-walled cysts, predominantly in the upper and middle lung fields with relative sparing of lung bases; 3) a marked predominance of alveolar macrophages in bronchoalveolar lavage, with no lymphocytosis and no pathogen; and 4) exclusion of alternative diagnoses [1,5]. The patients’ records were systematically reviewed to confirm the PLCH diagnosis at the time of inclusion.

All of the patients provided written informed consent. Additional details on the inclusion and exclusion criteria are provided in the Additional file 1.

### Follow-up

The patients were managed in an outpatient manner at each study centre. Study visits occurred at baseline and at three, six, 12, 18, and 24 months. The patients were strongly encouraged to stop smoking at inclusion and during all of the follow-up visits in the study, including the use of dedicated smoking cessation consultations at each participating centre. The prescription of medications used in the smoking cessation programs was left to the discretion of the physician investigators.

At each visit, clinical evaluation, smoking status (based on the patients’ self-reports and urinary cotinine measurements blinded for the patients) [13], lung function, blood gases, and 6-minute walk test results were recorded. The patients also completed the St George’s Respiratory Questionnaire (SGRQ) [14]. Lung HRCT and Doppler echocardiography (as a screening test for PH) [15] were performed every six months. The evaluations performed at each visit are detailed in the Additional file 1.

### Data collection

A standardised case report form was completed at each investigation centre. The data were monitored by independent clinical research assistants. All of the HRCT scans were centrally analysed by a radiologist (C de M) and a chest physician (AT), both of whom had no knowledge of the clinical or functional findings. Semi-quantitative nodular and cystic CT scores were calculated, and the patients were classified into subgroups according to the CT score values, as previously described [5]. The presence of other smoking-related lung abnormalities (ground glass opacities and emphysema) were also recorded [16,17]. During the follow-ups, an HRCT score variation of at least four points was considered significant. Additional details about the lung CT analyses and on the scoring that was used is provided in the Additional file 1.

### Endpoints

The primary outcome was the progression of PLCH, based on lung function deterioration, defined as a decrease of at least 15% in FEV_1_, forced vital capacity (FVC) and/or the diffusing capacity of carbon monoxide (DL_CO_) compared with the baseline values.

Additionally, because prolonged constitutional symptoms and the occurrence of multiple pneumothoraces were reportedly associated with poor outcomes of PLCH [18], patients presenting these features during their follow-up were also considered as having progressive disease, even in the absence of the deterioration of lung function.

Secondary outcomes included variations in the lung function parameters over time, the 6-minute walk test and blood gas results, HRCT findings, SGRQ scores, and the occurrence of PH.

### Statistical analysis

Descriptive statistics are presented, namely the mean ± standard deviation (SD) or median (interquartile range [IQR]) values.

The cumulative incidence of the lung function outcome over time was estimated using the non-parametric Kaplan-Meier method. Cox proportional hazards models with time-dependent covariates were used to calculate the cause-specific hazards ratio (HR) of lung function deterioration associated with smoking status, while fully adjusting for potential confounders. The time-varying smoking patterns were modelled with three covariates: 1) the subject’s smoking status (smoker or non-smoker) at the current visit; 2) the subject’s smoking status over the previous six or 12 months; and 3) tobacco non-smoker status during the entire study period.

Mixed models incorporating repeated measures over time on the same subjects (generally correlated) were used for the continuous outcome measurements (i.e., lung function parameters and HRCT scores).

All of the statistical analyses were performed using SAS 9.3 (SAS Inc., Cary, NC, USA) and R 3.0.2 (http://www.R-project.org/). Two-sided P-values less than 0.05 were considered significant.

## Results

### Study population

Sixty-three patients with PLCH were enrolled in the study. Five patients were excluded (three patients were immediately lost to follow-up after inclusion, one patient withdrew his informed consent, and one patient with lymphangioleiomyomatosis had been erroneously included).

The disease was isolated in the lung in all but two patients who had associated localised bone lesions. The diagnosis of PLCH was histologically confirmed in 21 patients (36%; surgical lung biopsy, n = 20; bone biopsy, n = 1). The median time between the diagnosis of PLCH and inclusion in the study was 3.8 months (IQR: 2–7 months). The characteristics of the patients at the time of inclusion are shown in Table 1. No superimposed lung HRCT ground glass opacities were observed, whereas emphysema was present in 5 patients (localised n = 3, diffuse n = 2, both with histologically proven PLCH).

**Table 1:** Baseline characteristics of the patients*

**Characteristic**	**N = 58**
Age, yrs	35.6 ± 10.8
Female sex, n (%)	31 (53)
Race, n (%)	
White	55 (95)
Other	3 (5)
Smoking history, pack-years	21 ± 17
At diagnosis, n (%)	
Current smokers	56 (97)
Ex-smokers	2 (3)
At inclusion, n (%)	
Current smokers	39 (67)
Clinical features, n (%)	
Asymptomatic	21 (36)
Cough	30 (52)
Dyspnoea	26 (45)
NYHA class II/III	23/2
History of pneumothorax	11 (19)
Constitutional symptoms†	6 (10)
Pulmonary function testing	
FEV_1_	
Volume, ml	2974 ± 839
% predicted	87 ± 18
FVC	
Volume, ml	3787 ± 1036
% predicted	93 ± 18
FEV_1_/FVC, %	75.5 ± 8.8
TLC, % predicted	100.6 ± 15.3
RV, % predicted	116.5 ± 36.2
RV/TLC, % predicted	114.4 ± 30.2
DL_CO,_ % predicted	64.3 ± 13.2
Normal lung function, n (%)	7 (12)
Restriction, n (%)‡	5 (9)
Obstruction, n (%)‡	15 (26)
Bronchial hyperreactivity, n (%)‡	6 (10)
DL_CO_ <80% predicted, n (%)‡	49 (87)
PaO_2,_ mm Hg	87 ± 10
6-Minute walk distance, m	514 ± 93
HRCT nodular score§	8 ± 4.5
Nodular score subgroup, n (%)	
Low	26 (46)
Intermediate	20 (36)
High	10 (18)
HRCT cystic score§	8.2 ± 5
Cystic score subgroup, n (%)	
Low	30 (54)
Intermediate	18 (32)
High	6 (11)
Very high	2 (3)
SGRQ score║	20.2 ± 18.8

*Definition of abbreviations*: *IQR* interquartile range, *NYHA* New York Heart Association, *FEV*_*1*_ forced expiratory volume in 1 second, *FVC* forced vital capacity, *TLC* total lung capacity, *RV* residual volume, *DL*_*CO*_ diffusing capacity for carbon monoxide, *PaO*_*2*_ arterial partial oxygen pressure, *HRCT* high-resolution computed tomography, *SGRQ* St George’s Respiratory Questionnaire.

*Plus-minus values are the means ± SDs.

†Constitutional symptoms were associated with respiratory symptoms in four of six patients.

‡Lung function restriction was defined as TLC <80% of the predicted value and obstruction as an FEV_1_/FVC ratio <70%. Bronchial hyperreactivity corresponded to a post-bronchodilator FEV_1_ improvement of >12% and >200 ml compared with the baseline values. The DL_CO_ was available for 56 patients.

§HRCT was available at inclusion for 56 patients. The maximal values for the HRCT nodular and cystic scores were 18 and 24, respectively.

║SGRQ was available at inclusion for 55 patients. The scores ranged from 0 to 100, with higher scores indicating worse functioning.

Doppler echocardiography was available at inclusion for 57 patients and none had criteria for PH. The median tricuspid regurgitant jet velocity was 2.4 m∙s^−1^ (IQR 2.3-2.5 m∙s^−1^), the median pulmonary arterial systolic pressure was 30 mm Hg (IQR 26–33 mm Hg), and no patient had increased dimensions of right heart chambers.

The patients were followed for a median of 24 months (IQR: 22–25 months). Fifty-five and 44 patients were evaluable at one and two years of follow-up, respectively. One patient incidentally died of myocardial infarction five months after inclusion, and 13 patients discontinued the study. No patients received systemic corticosteroids or immunosuppressive treatment during the study. A flow chart of the study is provided in the Additional file 1.

### Progression of PLCH

No patients complained of constitutional symptoms during the follow-up visits. Two patients experienced pneumothorax at one and 12 months after inclusion, respectively; pneumothorax spontaneously resolved in one case and was treated by surgical pleurodesis in the other. No patient progressed from isolated PLCH to multisystem disease.

**Figure 1:**
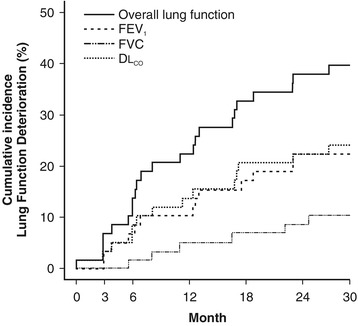
**The estimated cumulative incidence of lung function deterioration during the study.** The overall lung function corresponds to a decrease of at least 15% in FEV_1_, FVC, and/or DL_CO_. FEV_1_ = forced expiratory volume in 1 second; FVC = forced vital capacity; DL_CO_ = diffusion capacity for carbon monoxide.

Compared with their baseline values, 23 (40%) patients had a decrease of at least 15% in FEV_1_, FVC and/or DL_CO_ within a median of one year of follow-up (Table 2). More precisely, FEV_1_, FVC, and DL_CO_ decreased by at least 15% in 13 (22%) patients, six (10%) patients, and 14 (24%) patients, respectively. The two patients who had a pneumothorax during their follow-up had previously presented a decrease in their lung function. Figure 1 shows the cumulative incidences of the deterioration of lung function parameters during the study. The estimated cumulative incidence of deterioration at 24 months was 38% (95% CI: 25-51%) considering any functional parameter, 22% (95% CI: 11-33%) for FEV_1_ and DL_CO_ and 9% (95% CI: 1-16%) for FVC.

**Table 2:** Characteristics of the 23 patients with deteriorating lung function*

**Parameter (n)**	**Time of deterioration month**	**Baseline**	**Deterioration**	**Extent of deterioration**	
		**Absolute value†**		**Absolute value†**	**P value‡**
		**% of predicted**		**%**	
FVC (n = 6)	14.3 (8 · 4–23)	3350 (3100–3810)	2745 (2330–2950)	−665 (−770; −590)	0.03
		98 (85–112)	80 (64–94)	−20 (−22.6; −16.2)	
FEV_1_ (n = 13)	12.8 (5.8–18.2)	2540 (2390–3300)	1990 (1740–2770)	−460 (−530; −400)	<0.001
		90.1 (80; 96 · 0)	77.2 (67 · 9; 78 · 7)	−16.7 (−18.8; −15.8)	
DL_CO_ (n = 14)	11.7 (6.2–17.6)	6 (4.7-6.5)	4.82 (3.6-5.31)	−1.17 (−1 · 4; −0.95)	<0.001
		64.3 (57–72)	50 (44–60)	−17.8 (−19 · 2; −16 · 3)	
6-minute walk distance, m		505 (480–547)	529 (471–564)	0 (−36; +31.5)	0.71
PaO_2_, mm Hg		79 (75–88)	86 (79–90)	−1 (−6; +12)	0.38
SGRQ score§		21.8 (10 · 8–43 · 5)	16.1 (7.3–28.7)	−3.2 (−11.2; +2.1)	0.12
HRCT nodular score║		7.5 (6–10)	6.5 (5–10)	0 (0–0)	0.69
HRCT cystic score		6.5 (5–11)	7 (4–12)	0 (0–2)	0.18

*Definition of abbreviations*: *FVC* forced vital capacity, *FEV*_*1*_ forced expiratory volume in 1 second, *DL*_*CO*_ diffusion capacity for carbon monoxide, *PaO*_*2*_ the arterial partial oxygen pressure, *SGRQ* St George’s Respiratory Questionnaire, *HRCT* high-resolution computed tomography.

*Results are expressed as the medians and interquartile ranges (in parentheses). Lung function deterioration was defined as a decrease of at least 15% in the FEV_1_, FVC and/or DL_CO_.

†Absolute values are expressed in ml for FVC and FEV_1_ and in mmol/min/kPa for DL_CO_.

‡A paired *t*-test was used for the comparisons.

§SGRQ was available for 22 patients. Values ranged from 0 to 100, with higher scores indicating worse functioning.

║The maximal values for the HRCT nodular and cystic scores were 18 and 24, respectively.

### Patients with lung function deterioration

Table 2 summarises the characteristics of the patients with lung function deterioration. At the time of deterioration, 17 patients (74%) had a functional class of dyspnoea that was ≥2 according to the New York Heart Association (NYHA) criteria. Among the 13 patients with a decline in FEV_1_, nine (69%) had an obstructive pattern at the time of deterioration (FEV_1_: 66 ± 10.4% of the predicted value). Among the six patients who had decreases in their FVC, only one patient had a restrictive pattern (TLC: 70% of the predicted value), whereas the remaining five patients had a parallel increase in their residual volume (RV), resulting in a normal TLC (TLC: 112 ± 9.5% of the predicted value). Eight patients had an isolated decrease in DL_CO_ (56 ± 12% of the predicted value at the time of deterioration). Serial Doppler echocardiograms were available for 7 of these 8 subjects and showed criteria for likely PH in one patient at 18 months of follow-up (tricuspid regurgitant jet velocity: 3.34 m∙s^−1^; pulmonary arterial systolic pressure: 54 mm Hg). PH was confirmed by right heart catheterisation (mean pulmonary artery pressure: 29 mm Hg). The results of the 6-minute walk test did not significantly vary between baseline and the time of lung function deterioration.

### Smoking statuses of the patients during the study

**Figure 2:**
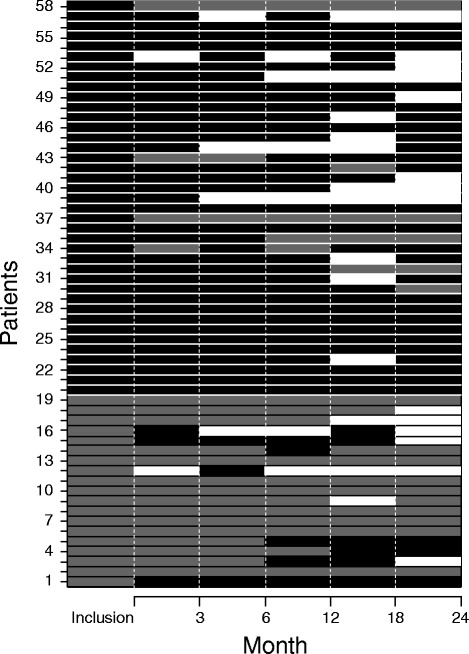
**Detailed changes in the smoking statuses of the patients during the study.** The patients’ smoking statuses were recorded at each scheduled visit based on their self-reports and urinary cotinine concentrations (except in patients using nicotine replacement therapy). Each line represents a patient. Periods of current smoking are displayed in black, periods of smoking cessation in grey, and periods of loss to follow-up in white.

The smoking statuses of the patients at each visit were based on their self-reports and their urinary cotinine measurements for all of the cases, except for one patient who was undergoing nicotine replacement therapy. Figure 2 shows the variations in the smoking statuses of the patients over time. At inclusion, 39 patients currently smoked, and 19 patients had stopped smoking between diagnosis and their inclusion in the study. Among these 19 non-smoking patients at inclusion, 11 patients remained non-smokers throughout their follow-ups in the study for a median duration of 24.2 months (IQR: 23–25 months), whereas eight patients resumed smoking for variable periods of time. Conversely, among the 39 patients who smoked at inclusion, three patients stopped smoking transiently during their follow-ups for three or six months, and five patients were weaned from tobacco for six (n = 1), 12 (n = 1), 18 (n = 1), or 24 (n = 2) months. Taken together, 13 (22%) patients remained weaned from tobacco throughout their follow-ups in the study.

Based on their lung function parameters, there was no difference in severity between non-smoking and smoking patients at the time of inclusion (p > 0.1).

### Factors predictive of lung function deterioration

Table 3 presents the detailed univariate analyses of the factors predictive of lung function deterioration. At inclusion, the factors that influenced the risk of lung function deterioration were older age, the presence of an airflow obstruction pattern, decreased PaO_2,_ and a high SGRQ score. Smoking status at inclusion was also associated with an increased risk of lung function deterioration. In contrast, FEV_1_ impairment at inclusion, the initial presence of air trapping (defined as an RV/TLC ratio of >120% of the predicted value), and HRCT cystic-score values were not associated with further deterioration in lung function. When considering the predictive factors jointly in a multivariable model, only smoking status and PaO_2_ at inclusion still influenced the hazard of lung function deterioration. Indeed, smoking at inclusion was associated with an increased hazard of progression (HR = 3.28; 95% CI: 1.00-11.1; p = 0.05); conversely, the higher the PaO_2_ level was at inclusion, the lower the risk was of progression (HR = 0.94, 95% CI: 0.90-0.98; p = 0.0036). Notably, among the patients who smoked at inclusion, those whose lung function deteriorated during follow-up were older (41.4 ± 11.3 yrs *vs*. 32.8 ± 7.6 yrs, p = 0.016), had lower PaO_2_ values (81.1 ± 9.1 mm Hg *vs.* 91.0 ± 7.6 mm Hg, p = 0.0013) and had somewhat higher SGRQ scores (26.2 ± 20.3 *vs*. 18.4 ± 18.5, p = 0.16) compared with those whose lung function did not deteriorate.

**Table 3:** Univariate analyses of the predictive factors (measured at inclusion) of lung function deterioration*

**Characteristic**	**Deterioration (n = 23)**	**No deterioration (n = 35)**	**HR (95% CI)**	**P value**
Demographic features				
Age, yrs	41.1 ± 12.0**	32.0 ± 8.2	1.7 (1.2-2.4)†	0.002
Sex, n (%)				
Male	12 (52)	15 (43)	1.0	
Female	11 (48)	20 (57)	0.98 (0.4-2.3)	0.97
Smoking status, n (%)				
Smokers	20 (87)	19 (54)	1.0	
Non-smokers	3 (13)	16 (46)	0.25 (0.1-0.85)	0.027
Clinical features, n (%)				
Asymptomatic	6 (26)	15 (43)	0.6 (0.2-1.7)	0.38
Cough	10 (48)	10 (34)	1.3 (0.5-3.2)	0.54
Dyspnoea	13 (57)	12 (34)	2.0 (0.8-4.6)	0.11
Lung function parameters				
FEV_1_				
% predicted	85.9 ± 13.7	87.8 ± 20.4	0.9 (0.7-1.2)†	0.45
≤59% predicted, n (%)	5 (14)	1 (4)	1 · 0	
60-79% of predicted	7 (20)	6 (26)	2.8 (0.3-23.3)	0.35
≥80% predicted	23 (66)	16 (70)	2.6 (0.3-19.7)	0.36
FVC				
% predicted	92.4 ± 19.6	93.4 ± 17.1	0.9 (0.7-1.2)†	0.92
TLC				
% predicted	101.0 ± 18.0	100.3 ± 13.4	1.0 (0.8-1.3)†	0.98
Restriction, n (%)‡	3 (13)	2 (6)	1.9 (0.5-6.4)	0.32
RV				
% predicted	118.8 ± 33.4	115.0 ± 38.4	1.0 (0.9-1.1)†	0.67
RV/TLC	36.1 ± 9.6	32.0 ± 9.6	1.0 (1.0-1.02)	0.58
Air trapping, n (%)‡	10 (43)	11 (32)	1.7 (0.7-3.9)	0.23
FEV_1_ /FVC	74.1 ± 9.8	76.5 ± 8.1	0.98 (0.9-1.0)	0.42
Airflow obstruction, n (%)‡	10 (43)	5 (14)	2.9 (1.3-6.8)	0.014
DL_CO_				
% predicted	63.8 ± 12.9	64.6 ± 13.2	0.3 (0.0-9.3)†	0.50
<72% predicted, n (%)	17 (74)	24 (69)	1.0	
≥72% predicted	6 (26)	11 (31)	0.7 (0.3-1.7)	0.42
6-Minute walk distance, m				
% of the predicted value	78.7 ± 14.0	73.2 ± 10.1	1.4 (0.9-2.0)†	0.09
PaO_2_	81.4 ± 9.7	90.8 ± 9.0	0.94 (0.91-0.98)†	0.0014
Lung HRCT§				
Nodular score	8.4 ± 4.8	7.8 ± 4.3	1.1 (0.7-2.0)║	0.46
Cystic score	8.0 ± 3.9	8.4 ± 5.7	1.0 (0.7-1.4)║	0.89
SGRQ score	25.7 ± 20.1	15.1 ± 16.8	1.3 (1.1-1.6)†	0.012

*Definition of abbreviations*: *HR* hazard ratio, *CI* confidence interval, *FEV*_*1*_ forced expiratory volume in 1 second, *FVC* forced vital capacity, *TLC* total lung capacity, *RV* residual volume, *DL*_*CO*_ diffusion capacity for carbon monoxide, *PaO*_*2*_ arterial partial oxygen pressure, *HRCT* high-resolution computed tomography, *SGRQ* St George’s ;Respiratory Questionnaire.

*Lung function deterioration was defined by a decrease of at least 15% in FEV_1_, FVC and/or DL_CO_ compared with the baseline values.

**Plus-minus values are the means ± SDs.

†Reported HRs are given for an increase of 10 units.

‡Lung function restriction was defined as a TLC <80% of the predicted value, air trapping as an RV/TLC ratio >120% of the predicted value and obstruction as an FEV_1_/FVC ratio <70%.

§HRCT was available at inclusion for 56 patients (22 and 34 patients in each group). The maximal values for the HRCT nodular and cystic scores were 18 and 24, respectively.

║Reported HRs are given for an increase of 4 points.

¶SGRQ was available for 55 patients at inclusion (23 and 32 patients in each group). The scores ranged from 0 to 100, with higher scores indicating worse functioning.

### Lung function outcomes according to smoking status of the patients

Table 4 presents the estimated effects of the patients’ smoking statuses (at inclusion and over time) on lung function deterioration. Tobacco use over time was associated with an increased hazard of pulmonary function deterioration. Conversely, smoking discontinuation during the study was associated with a decreased risk of subsequent lung function deterioration, even for patients who had stopped smoking during the previous six months. Moreover, these effects of smoking status remained statistically significant when adjusting for the potential confounder identified by the multivariate prognostic analysis (i.e., PaO_2_ at inclusion). In contrast, smoking status over time was not associated with variations in the HRCT scores (data not shown).

**Table 4:** Estimated effects of the smoking status at baseline and over time on the hazard of subsequent lung function deterioration*

**Smoking status**	**HR (95** **%** **CI) Unadjusted**	**P value**	**HR (95** **%** **CI)**	**P value Adjusted†**
Baseline non-smoking	0.25 (0.07-0.85)	0.027	0.30 (0.09-1.00)	0.05
Time-dependent non-smoking	0.25 (0.08-0.97)	0.04	0.34 (0.10-1.14)	0.08
No smoking during the past six months	0.25 (0.07-0.84)	0.025	0.29 (0.08-0.97)	0.044
No smoking during the past 12 months	0.23 (0.07-0.79)	0.020	0.28 (0.08-0.97)	0.045
No smoking during the study period	0.22 (0.06-0.73)	0.014	0.28 (0.08-0.94)	0.040

*Definition of abbreviations*: *FEV*_*1*_ forced expiratory volume in 1 second, *FVC* forced vital capacity, *DL*_*CO*_ diffusion capacity for carbon monoxide, *HR* hazard ratio, *CI* confidence interval.

*Lung function deterioration was defined as a decrease of at least 15% in FEV_1_, FVC and/or DL_CO_ compared with the baseline values.

†The adjustment predictive factor at inclusion was baseline PaO_2_, as selected by the multivariable prognostic model.

### Secondary outcomes of the study

Considering the entire study population, the mean yearly decline in lung function parameters of the entire population was 68 ± 90 ml (1.2 ± 1.8%) for FEV_1_, 43 ± 107 ml (0.1 ± 1.7%) for FVC and 0.24 ± 0.2 mmol/min/kPa (1.4 ± 1.5%) for DL_CO_. However, at the last follow-up, 24 (41%) patients had an airflow obstruction (FEV_1_: 68.4 ± 14.5% of the predicted value), and five (9%) patients exhibited a restrictive pattern (TLC: 73.6 ± 4.5% of the predicted value). Notably, these patients did not experience pneumothoraces nor have other smoking-related interstitial lung disease features (i.e., ground glass opacities) superimposed on their lung HRCT during during their follow-up. Six patients had a PaO_2_ of <70 mm Hg (61 ± 10 mm Hg) at the last evaluation.

Among the 52 patients who had serial Doppler echocardiograms, with the exception of the patient described above, no other patient exhibited criteria for PH.

**Figure 3:**
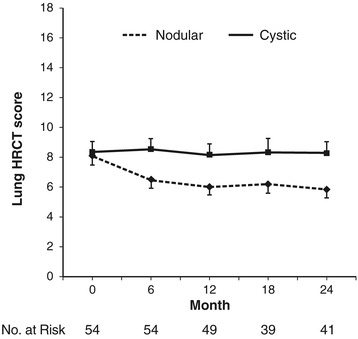
**Variations in the lung HRCT scores at the different scheduled visits of the study.** The data are expressed as the means ± SEMs of the lung HRCT nodular and cystic scores. The lung HRCT was available at inclusion for 56 patients. The maximal values for the HRCT nodular and cystic scores are 18 and 24, respectively.

Figure 3 displays the variations in the lung HRCT scores during the study. At 24 months, the cumulative incidence of cystic score deterioration was estimated to be 10.7% (95% CI: 2.5-19%). Additional details about the variations in the secondary outcomes over time are provided in the Additional file 1.

## Discussion

This multicentre, prospective study evaluated the early outcome of a homogeneous cohort of patients with untreated PLCH. We were able to demonstrate that 1) a substantial proportion of patients experienced an important decline in their lung function parameters within two years of follow-up and 2) the smoking status of the patients was associated with the risk of subsequent lung function deterioration.

All of the subjects had recent PLCH, as evidenced by the high percentage of initially asymptomatic patients and the low impairment of lung function at inclusion. Similarly, the HRCT findings were also characteristic of recent disease because all of the patients had nodular lesions, whereas less than 15% had high HRCT cystic scores [5,19].

Considering the entire study population, lung function varied weakly during the study period, as exemplified by the mean yearly decline in lung function parameters. Strikingly, however, 40% of the patients presented a decrease of at least 15% in FEV_1_, FVC and/or DL_CO_ within a median of one year of follow-up. Although the decreases in FVC, FEV_1,_ and DL_CO_ were equally significant, decreases were more frequent for the last two parameters. The magnitude of the FEV_1_ decrease in this subgroup of patients was beyond that observed in currently smoking COPD patients [20], and thus probably reflected bronchiolar damage caused by LCH lesions [2,3].

In accordance with the results of retrospective studies, we found, in univariate analyses, that older age and the presence of airflow obstruction at inclusion were associated with worse outcomes [5,11,18,21]. In the present study, decreased PaO_2_ and an altered respiratory quality of life, as assessed by the SGRQ, were also associated with an increased risk of lung function deterioration. In contrast, we did not confirm that a decrease in FEV_1_ or DL_CO_ or the presence of air trapping was associated with a poor outcome [5,11,18,21]. These differences were most likely due to the heterogeneity of the patients included in the retrospective studies, particularly concerning the duration of their disease [5,11,18,21]. Furthermore, in the multivariate analysis, only baseline PaO_2_ remained associated with disease progression. We hypothesize that the reason behind this correlation was that lower PaO_2_ may reflect more impaired gas exchange in the lung and/or pulmonary vascular involvement in some patients. Finally, the 6-minute walk test was not associated with lung function deterioration.

Based on both self-reports and blinded urinary cotinine measurements, the smoking status of the patients markedly changed over time, and only a minority (approximately 20%) were weaned from tobacco during the study period. The significant variations in smoking status over time most likely explained the discrepancies reported in previous studies concerning the effects of smoking on the outcomes of the disease [4,5,7-12]. Here, using appropriate, time-dependent Cox models, we formally showed that a non-smoker status was associated with a decreased risk of subsequent lung-function deterioration, even in patients who stopped smoking for at least six months during follow-up. Importantly, this effect on lung function outcome persisted even after adjusting for PaO_2_ at inclusion. Of note, no differences in the severity of baseline lung function were observed between non-smoking and smoking patients. In contrast, smoking status over time was not associated with variations in HRCT findings.

Airflow obstruction was the predominant lung function profile observed in the entire study population, whereas true lung restriction, based on the TLC measured by plethysmography, was observed in less than 10% of the patients. Notably, these patients did not experience pneumothoraces nor have other smoking-related interstitial lung disease features (i.e., ground glass opacities) superimposed on their lung HRCT during their follow-up. By serial plethysmography, patients with a decreased FVC had a parallel increase in their RV, as previously observed in bronchiolar disorders [22]. Although PH has primarily been described in patients with longstanding PLCH [23-25], we found here that such complications may rarely occur earlier in the disease course.

Finally, whereas constitutional symptoms and the occurrence of pneumothoraces were reportedly associated with poor outcomes of PLCH [18], we did not observe constitutional symptoms during follow-up in patients with isolated lung involvement, whereas pneumothoraces occurred rarely during follow-up.

This study has some limitations. Although recruiting a cohort of 58 patients with newly diagnosed PLCH over a period of three years was a challenge, given the size of the cohort, one could fail to identify factors with a minor influence on the outcome of the disease.

## Conclusions

Because lung function can deteriorate early during the course of recent PLCH in a substantial proportion of patients and is difficult to predict in an individual patient, it is important to evaluate patients serially using FEV_1_, FVC, and DL_CO_ measurements on a three- to six-month basis. An isolated decreased DL_CO_ should prompt screening for PH by Doppler echocardiography. Whereas lung HRCT is essential for diagnosis, systematic close sequential lung HRCTs are of limited value. The 6-minute walk test appears to be less informative than in patients with advanced disease [25]. Given the strong tobacco addictions of patients with PLCH, robust efforts should be undertaken to include these patients in smoking cessation programs.

## Additional file

Additional file 1: **Supplementary material.** Supplementary methods, results (**Table S1**) and figure legends. **Figure S1A.** Flow chart of the study. **Figure S1B.** Visit calendar of the study. **Figure S2.** Patient distribution among the subgroups based on lung HRCT nodular (Panel A) and cystic scores (Panel B) during the study.

## Abbreviations

CI: Confidence interval
DL_CO_: Diffusion capacity for carbon monoxide
FEV_1_: Forced expiratory volume in 1 second
FVC: Forced vital capacity
HR: Hazard ratio
HRCT: High-resolution computed tomography
IQR: Interquartile range
LCH: Langerhans cell histiocytosis
NYHA: New York Heart Association
PaO_2_: Arterial oxygen partial pressure
PH: Pulmonary hypertension
PLCH: Pulmonary Langerhans cell histiocytosis
RV: Residual volume
SD: Standard deviation
SGRQ: St George’s Respiratory Questionnaire
TLC: Total lung capacity
