# Supplementary material for: The natural history of adult pulmonary Langerhans cell histiocytosis: a prospective multicentre study
Source: Orphanet J Rare Dis. 2015 Mar 14;10:30. doi: 10.1186/s13023-015-0249-2 (PMC4438520; doi:10.1186/s13023-015-0249-2)
Supplement: Supplementary file 1 — Supplementary material. Supplementary methods, results (Table S1) and figure legends. Figure S1A. Flow chart of the study. Figure S1B. Visit calendar of the study. Figure S2. Patient distribution among the subgroups based on lung HRCT nodular (Panel A) and cystic scores (Panel B) during the study. [file 13023_2015_249_MOESM1_ESM.zip › 13023_2015_249_add1/7530303251528596_add1.pdf]

Online supplementary material

The natural history of adult pulmonary Langerhans cell histiocytosis: a prospective multicentre study

Abdellatif Tazi MD, PhD, Constance de Margerie MD, Jean Marc Naccache MD, Stéphanie Fry MD, Stéphane Dominique MD, Stéphane Jouneau MD, PhD, Gwenaël Lorillon MD, Emmanuelle Bugnet PhD, Raphael Chiron MD, Benoit Wallaert MD, Dominique Valeyre MD, and Sylvie Chevret MD, PhD

Methods

Lung Function

Lung volumes were evaluated by plethysmography, and FEV_1 and FVC were determined by flow curve volume. DL_{CO} was measured using the single-breath method. Predictive values were determined according to the European Respiratory Society criteria [1]. Obstruction was defined as a ratio of FEV_1 to FVC <70% [2], lung restriction was defined as a TLC <80% of the predicted value, and air trapping was defined as a ratio of RV to TLC >120% of the predicted value. FEV_1 was measured both at baseline and after four separate doses of 100 µg of inhaled salbutamol. An increase in FEV_1 of >12% and >200 ml compared with the baseline measurement defined bronchial hyper-responsiveness [3]. Blood gases were obtained by puncture of the radial artery with the patient resting and breathing room air.

Six-minute Walk Distance

The six-minute walk test was performed indoors along a flat and straight 30-m course and was supervised by a trained nurse, according to the American Thoracic Society guidelines [4]. The distance walked, Borg dyspnoea index, New York Heart Association (NYHA) functional class and oxygen saturation were measured at rest and after 6 min of exercise.

St. George's Questionnaire (SGRQ)

The standardised form of the SGRQ was completed by the patient at each visit, except for the first section, which concerned the respiratory symptoms over the previous 12 months because only patients recently diagnosed with pulmonary Langerhans cell histiocytosis (LCH) were included in the study. The calculation of the score was performed as previously described, using a zero value for the first section [5]. The scoring values could range from 0 to 100, with higher scores indicating worse functioning values [5].

Lung HRCT Analysis and Semi-quantitative Nodular and Cystic Scores

Lung CT was obtained with different multidetector helical scanners, during breath holding at the end of inspiration in patients lying in the supine position. All of the patients underwent HRCT, with a reconstructed section thickness of 1 mm or less and a high-spatial frequency algorithm. The images were obtained at window levels appropriate for the pulmonary parenchyma (width: 600-1600 HU).

Each lung was divided into three areas, from the lung apices to the domes of the diaphragm: the upper level extended from the lung apex to the carina, the middle level extended between the carina and the lower pulmonary veins, and the lower level extended from below the lower pulmonary veins to the diaphragm. Each zone was assessed for the presence, type and extent of lung abnormalities.

The following values were attributed to the profusion of nodular lesions (including micronodules, nodules, and cavitary nodules): 0 (no nodules), 1 (mild), 2 (moderate), and 3 (diffuse nodules). The global nodular CT score was obtained by adding the values of the nodular scores for the six defined lung zones. Thus, the nodular HRCT score for the entire lung could range from 0 to a maximum value of 18 [6].

The extent of cystic lesions (including thick- and thin-walled cysts) was assessed for each of the defined lung areas on HRCT and was classified as a percentage of the lung surface analysed: 0 (no cyst); 1 (<25%); 2 (25-49%); 3 (50-75%); and 4 (>75%). For the whole lung, the maximum value of the cystic HRCT score was 24 [6].

The patients were divided into three categories based on their HRCT nodular scores — low (0-6), moderate (7-12), and high (13-18) — and into four subgroups to reflect the extent of their lung cystic lesions — low (≤6), moderate (7-12), high (13-18), and very high (19-24) HRCT cystic scores [6].

Because the HRCT lesions in pulmonary LCH are predominantly found in the upper and middle levels of the lungs [7], a 4-point variation corresponded, on average, to an increase or

decrease in the extent of the nodular or cystic lesions of at least one point in the upper and middle levels of both lungs and was thus considered significant.

The presence of other lung HRCT smoking related abnormalities, i.e., ground glass opacities and emphysema (presence and extent) was also recorded [8,9].

Doppler Echocardiography

Transthoracic echocardiography was used in all of the patients to screen for pulmonary hypertension (PH), and it was performed by a referent cardiologist in each centre participating in the study. The continuous-wave Doppler sampling of the peak tricuspid-valve regurgitant jet velocity was used to calculate the pulmonary arterial systolic pressure, as previously described [10]. The dimensions of the right heart chambers were also systematically measured. Based on the ECS/ERS guidelines, a tricuspid regurgitation velocity >3·4 $m.s^{-1}$ and/or systolic pulmonary artery pressure >50 mm Hg on Doppler echocardiography were considered suggestive of likely pulmonary hypertension [11]. In such cases, right heart catheterisation was performed to confirm the presence of pulmonary hypertension (defined as a mean arterial pulmonary pressure ≥25 mm Hg) [11].

Urinary cotinine, the major nicotine metabolite, was measured in urine samples that were collected at each visit in a blinded manner from the patients. The results, which reflect the last three or four days of exposure to tobacco smoke [12], were used as a surrogate to define the smoking statuses of patients who declared themselves non-smokers [13]. Depending on the centre, the urinary cotinine concentrations were measured by high-pressure liquid chromatography (HPLC) (smoker urinary cotinine concentrations >0·3 µmol/l) [14], liquid chromatography combined with mass spectrometry detection (LC-MS) (smoker urinary cotinine concentrations >0·28 µmol/l) [15] or the DRI ® Cotinine immunoassay (Microgenics) (smoker urinary cotinine concentrations >2·84 µmol/l) [16]. The urinary cotinine levels were not considered in patients using nicotine replacement therapy.

Study Protocol

This multicentre, prospective, observational cohort study was performed from May 2006 to May 2011, with an inclusion period of three years (2006-2009) and a follow-up duration of two years.

The exclusion criteria were as follows:

- age <18 or >75 years old;
- deeply impaired lung function: TLC <60% of the predicted value, FEV_1 <30% of the predicted value, PaO_2 <60 mm Hg breathing room air or patients under long-term oxygen or exhibiting signs of cor pulmonale;
- pregnant women or women of childbearing age not using contraception;
- extra-pulmonary localisations of LCH, except for localised bone lesions;
- requirement for systemic corticosteroid or immunosuppressant treatments for any reason subsequent to the diagnosis of pulmonary LCH and during the entire follow-up period in the study; and
- pulmonary LCH diagnosed for more than 48 months.

At the diagnosis, information concerning the demographic features of the patients (i.e., age, sex, ethnicity, smoking habits, and consumption of tobacco in pack-years) was collected. At each scheduled visit, the following clinical items were recorded: self-reported smoking habits, the dates of smoking discontinuation and eventual smoking resumption, the use of nicotine replacement therapy or other medications used for smoking cessation, the presence or absence and the date of respiratory signs (i.e., chest pain, cough, dyspnoea, haemoptysis, wheezing, crackles, digital clubbing, and cor pulmonale), the presence of pneumothorax, constitutional symptoms (e.g., temperature, night sweats, and body weight changes), and extrathoracic manifestations of LCH. At each visit, the patients were asked about the occurrence of any adverse events or any medication consumption since the previous visit.

The investigations performed at each visit during the study are shown in Figure E1.

References

1. Standardized lung function testing. Official statement of the European Respiratory Society. Eur Respir J Suppl 1993, 16:1-100.
2. Pauwels RA, Buist AS, Calverley PM, Jenkins CR, Hurd SS, GOLD Scientific Committee: Global strategy for the diagnosis, management, and prevention of chronic obstructive pulmonary disease. NHLBI/WHO Global Initiative for Chronic obstructive Lung disease (gold) workshop summary. Am J Respir Crit Care Med 2001, 163:1256-76.
3. Pellegrino R, Viegi G, Brusasco V, Crapo RO, Burgos F, Casaburi R, et al. Interpretative strategies for lung function tests. Eur Respir J 2005, 26:948-68.
4. ATS Committee on Proficiency Standards for Clinical Pulmonary Function Laboratories: ATS statement: guidelines for the six-minute walk test. Am J Respir Crit Care Med 2002, 166:111-7.
5. Jones PW, Quirk FH, Baveystock CM: The St George's respiratory questionnaire. Respir Med 1991, 85 Suppl B:25-31.
6. Tazi A, Marc K, Dominique S, de Bazelaire C, Crestani B, Chinet T, et al. Serial computed tomography and lung function testing in pulmonary langerhans' cell histiocytosis. Eur Respir J 2012, 40:905-12.
7. Brauner MW, Grenier P, Mouelhi MM, Mompoint D, Lenoir S: Pulmonary histiocytosis X: Evaluation with high-resolution CT. Radiology 1989, 172:255-8.
8. Caminati A, Harari S: Smoking-related interstitial pneumonias and pulmonary langerhans cell histiocytosis. Proc Am Thorac Soc 2006, 3:299-306.
9. Vassallo R, Jensen EA, Colby TV, Ryu JH, Douglas WW, Hartman TE, et al. The overlap between respiratory bronchiolitis and desquamative interstitial pneumonia in pulmonary langerhans cell histiocytosis: high-resolution CT, histologic, and functional correlations. Chest 2003, 124:1199-205.

10. Berger M, Haimowitz A, Van Tosh A, Berdoff RL, Goldberg E: Quantitative assessment of pulmonary hypertension in patients with tricuspid regurgitation using continuous wave Doppler ultrasound. J Am Coll Cardiol 1985, 6:359-65.
11. Task Force for Diagnosis and Treatment of Pulmonary Hypertension of European Society of Cardiology (ESC), European Respiratory Society (ERS), International Society of Heart and Lung Transplantation (ISHLT), Galiè N, Hoeper MM, Humbert M, Torbicki A, Vachiery JL, Barbera JA, et al. Guidelines for the diagnosis and treatment of pulmonary hypertension. Eur Respir J 2009, 34:1219-63.
12. Benowitz NL: Cotinine as a biomarker of environmental tobacco smoke exposure. Epidemiol Rev 1996, 18:188-204.
13. Gariti P, Alterman AI, Ehrman R, Mulvaney FD, O'Brien CP: Detecting smoking following smoking cessation treatment. Drug Alcohol Depend 2002, 65:191-6.
14. Ceppa F, El Jahiri Y, Mayaudon H, Dupuy O, Burnat P: High-performance liquid chromatographic determination of cotinine in urine in isocratic mode. J Chromatogr B Biomed Sci Appl 2000, 746:115-22.
15. Pacifici R, Pichini S, Altieri I, Rosa M, Bacosi A, Caronna A, et al. Determination of nicotine and two major metabolites in serum by solid-phase extraction and high-performance liquid chromatography, and high-performance liquid chromatography-particle beam mass spectrometry. J Chromatogr 1993, 612:209-13.
16. Van Vunakis H, Gjika HB, Langone JJ: Radioimmunoassay for nicotine and cotinine. IARC Sci Publ 1987, 81:317-30.

TABLE S1. Detailed variations of the secondary outcomes of the study during follow-up*

Characteristic	Inclusion	3 months	6 months	12 months	18 months	24 months
Number of Patients	(n=58)	(n=55)	(n=55)	(n=51)	(n=45)	(n=43)
TLC, % predicted	100 (91-109)	100 (92-110)	103 (93-111)	103 (96-108)	99 (95-107)	102 (93-109)
FVC, % predicted	94 (85-104)	96 (82-104)	96 (83-102)	96 (81-104)	93 (82-100)	84 (82-105)
RV, % predicted	110 (91-138)	109 (92-129)	114 (92-138)	107 (92-130)	106 (91-139)	115 (91-129)
RV/TLC, % predicted	109 (94-124)	104 (97-119)	111 (93-123)	106 (90-118)	108 (92-129)	112 (95-124)
FEV_1, % predicted	91 (75-98)	89 (74-98)	86 (76-97)	86 (74-98)	84 (69-97)	87 (69-96)
FEV1/FVC, %	75 (69-82)	75 (71-82)	74 (69-80)	75 (69-80)	74 (66-78)	75 (66-79)
DL_{CO}, % predicted†	64 (56-73)	62 (53-74)	62 (55-73)	62 (54-74)	57 (51-73)	61 (51-74)
PaO_2, mm Hg	87 (78-98)	92 (82-99)	91 (80-98)	93 (80-100)	88·5 (82·5-99)	89 (80-100)
6-Minute walk distance, m	502 (453-586)	510 (429-577)	529 (470-580)	511 (471-600)	499 (455-570)	520 (469-579)
HRCT nodular score‡	7·5 (4-10)	NA	6 (4-9·5)	6 (4-8)	6 (4-7)	6 (4-7)

HRCT cystic score‡	6<br>(5-11)	NA	7<br>(5-12)	6<br>(5-12)	6<br>(5-12)	6<br>(5-11)
SGRQ score§	14<br>(7-29)	14<br>(6-25)	13<br>(3-26)	8<br>(4-18)	9<br>(2-28)	14<br>(2-25)

Definition of abbreviations: TLC = total lung capacity; FVC = forced vital capacity; RV = residual volume; FEV_1 = forced expiratory volume in 1 second; DL_{CO} = diffusion capacity for carbon monoxide; PaO_2, = arterial oxygen partial pressure; HRCT = high-resolution computed tomography; SGRQ = St George's Respiratory Questionnaire; NA = not applicable*Results are expressed as the medians and interquartile ranges. †DL_{CO} was available for 56 patients at inclusion.‡Lung HRCT was available for 56 patients at inclusion and for 54, 49, 39, and 41 patients at 6, 12, 18, and 24 months, respectively. The maximal values for the HRCT nodular and cystic scores were 18 and 24, respectively.§ SGRQ scores were available for 55 patients at inclusion and for 53, 52, 48, 41 and 36 patients at 3, 6, 12, 18, and 24 months, respectively. Values ranged from 0 to 100, with higher scores indicating worse functioning.

Figure Legends

Figure S1. Flow chart (Panel A) and visit calendar of the study (Panel B).

* Urinary cotinine measurements were blinded for the patients.

Figure S2. Patient distribution among the subgroups based on lung HRCT nodular (Panel A) and cystic scores (Panel B) during the study.The maximal values for the HRCT nodular and cystic scores were 18 and 24, respectively. The patients were divided into three subgroups based on their HRCT nodular scores — low (0-6), moderate (7-12), and high (13-18) — and into four subgroups to reflect the extent of their lung cystic lesions — low (≤6), moderate (7-12), high (13-18), and very high (19-24) HRCT cystic scores.
