# Supplementary figures and images for: The natural history of adult pulmonary Langerhans cell histiocytosis: a prospective multicentre study
Source: Orphanet J Rare Dis. 2015 Mar 14;10:30. doi: 10.1186/s13023-015-0249-2 (PMC4438520; doi:10.1186/s13023-015-0249-2)

A

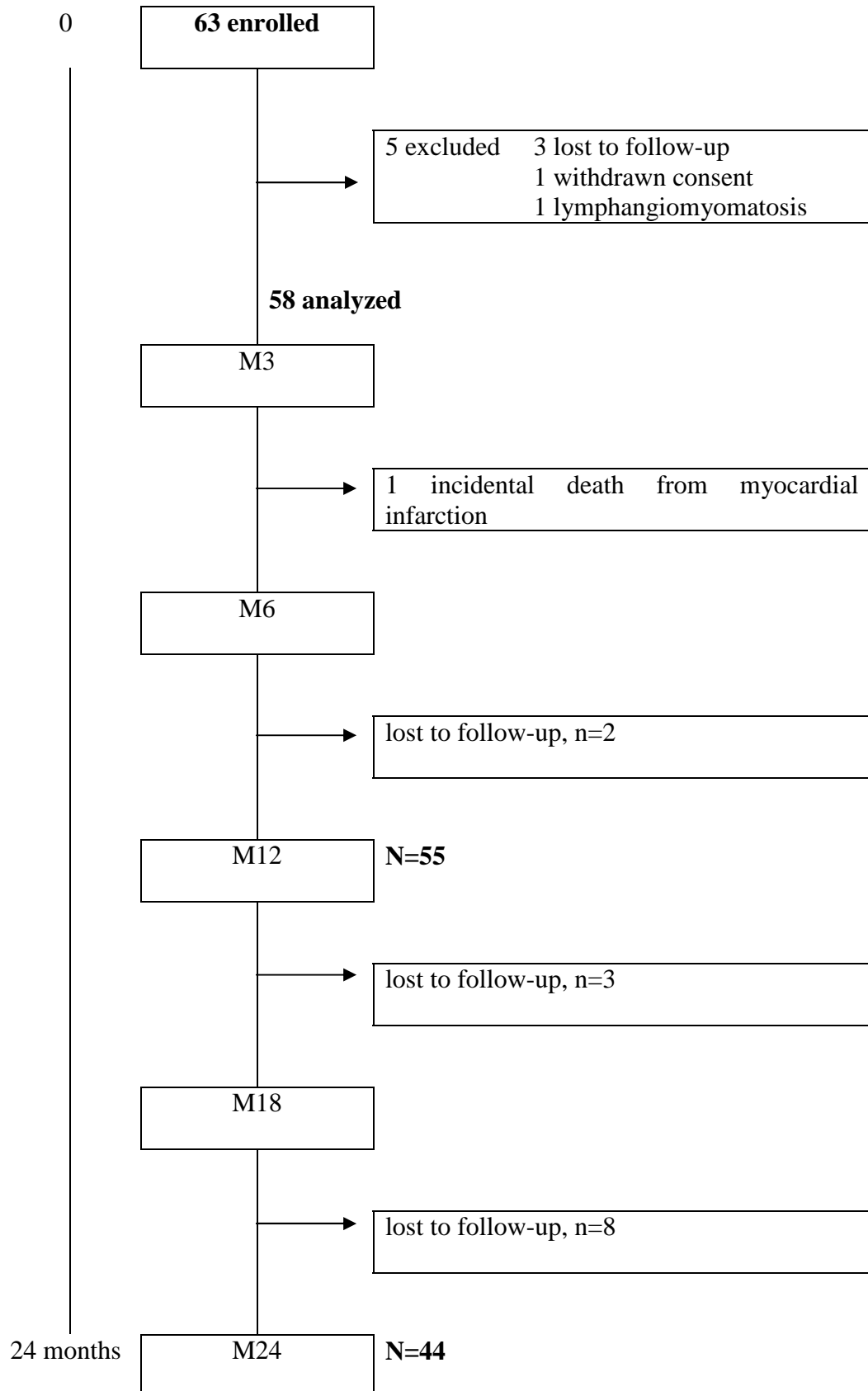

Supplement: Supplementary file 1 — Supplementary material. Supplementary methods, results (Table S1) and figure legends. Figure S1A. Flow chart of the study. Figure S1B. Visit calendar of the study. Figure S2. Patient distribution among the subgroups based on lung HRCT nodular (Panel A) and cystic scores (Panel B) during the study. [file 13023_2015_249_MOESM1_ESM.zip › 13023_2015_249_add1/7530303251528596_add2.pdf]

**A**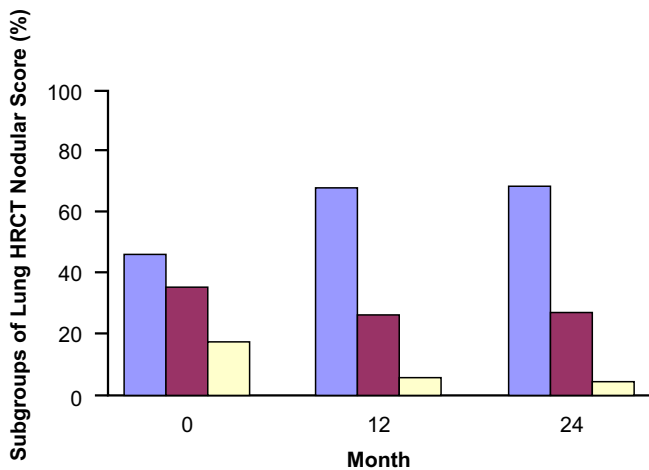**B**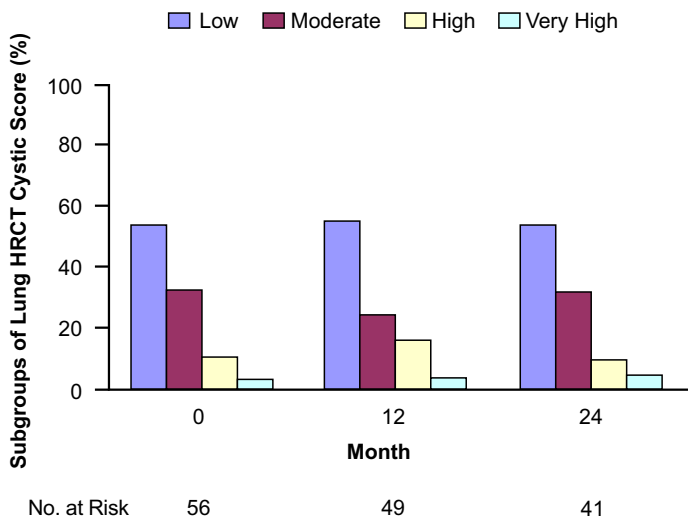

Supplement: Supplementary file 1 — Supplementary material. Supplementary methods, results (Table S1) and figure legends. Figure S1A. Flow chart of the study. Figure S1B. Visit calendar of the study. Figure S2. Patient distribution among the subgroups based on lung HRCT nodular (Panel A) and cystic scores (Panel B) during the study. [file 13023_2015_249_MOESM1_ESM.zip › 13023_2015_249_add1/7530303251528596_add4.pdf]
